# Functional Interplay Between Collagen Network and Cell Behavior Within Tumor Microenvironment in Colorectal Cancer

**DOI:** 10.3389/fonc.2020.00527

**Published:** 2020-04-30

**Authors:** Cuong Cao Le, Amar Bennasroune, Benoit Langlois, Stéphanie Salesse, Camille Boulagnon-Rombi, Hamid Morjani, Stéphane Dedieu, Aline Appert-Collin

**Affiliations:** ^1^Université de Reims Champagne-Ardenne, Reims, France; ^2^CNRS UMR 7369, Matrice Extracellulaire et Dynamique Cellulaire, MEDyC, Reims, France; ^3^Unité BioSpecT, EA7506, Reims, France; ^4^Laboratoire de Biopathologie, Centre Hospitalier Universitaire et Faculté de Médecine, Reims, France

**Keywords:** colorectal cancer, collagen, cancer-associated fibroblast, tumor cell, endothelial cell, *in vitro* model

## Abstract

Colorectal cancer is the second most common cancer diagnosed in men and the third most commonly occurring in women worldwide. Interactions between cells and the surrounding extracellular matrix (ECM) are involved in tumor development and progression of many types of cancer. The organization of the ECM molecules provides not only physical scaffoldings and dynamic network into which cells are embedded but also allows the control of many cellular behaviors including proliferation, migration, differentiation, and survival leading to homeostasis and morphogenesis regulation. Modifications of ECM composition and mechanical properties during carcinogenesis are critical for tumor initiation and progression. The core matrisome consists of five classes of macromolecules, which are collagens, laminins, fibronectin, proteoglycans, and hyaluronans. In most tissues, fibrillar collagen is the major component of ECM. Cells embedded into fibrillar collagen interact with it through their surface receptors, such as integrins and discoidin domain receptors (DDRs). On the one hand, cells incorporate signals from ECM that modify their functionalities and behaviors. On the other hand, all cells within tumor environment (cancer cells, cancer-associated fibroblasts, endothelial cells, and immune cells) synthesize and secrete matrix macromolecules under the control of multiple extracellular signals. This cell-ECM dialog participates in a dynamic way in ECM formation and its biophysical and biochemical properties. Here, we will review the functional interplay between cells and collagen network within the tumor microenvironment during colorectal cancer progression.

## Collagen and Colorectal Cancer: State of Play

In recent decades, several works have underlined the importance of the microenvironment in colon cancer progression (1). In the tumor microenvironment (TME), extracellular matrix (ECM) plays a key role in this process. Among ECM adhesive components, type I collagen is one of the important factors regulating cancer-related events at different tumorigenesis stages (2). After effacement of the basement membrane, paracrine signals from the nascent tumor lead to profound reorganizations of submucosal ECM that include deposition of fibrillar collagens together with growth factors and ECM-modifying enzymes that stimulate active vascular remodeling. Some recent studies based on global transcriptomic or proteomic approaches shed new light on the specific markers that are dysregulated during early steps of colon carcinogenesis, but also in locally advanced or metastatic colorectal cancer (CRC) (3–5). Interestingly, proteomic analysis of detergent insoluble fractions of paired primary colon tumors and liver metastasis compared with adjacent non-tumorous tissues illustrated the pathological samples' specific enrichment in core matrisome and several collagen-modifying enzymes such as MMPs, ADAMs, and LOXL1 (5). Desmoplasia and collagen deposition constitute a hallmark of CRC and various collagens including type I, VI, VII, VIII, X, XI, and XVIII were found accumulated in CRC samples (6–12). A recent study showed an increase of type I collagen in tumor tissues compared to normal tissue (13). Moreover, type I collagen mRNAs were also reported as increased in blood of CRC patients compared to healthy individuals (13, 14). Consistently, second harmonic generation imaging of fibrillar collagen contents has shown clinical efficacy to stratify high-grade tumors and relevance to predict CRC patient outcome (7, 15).

The most studied type I collagen receptors are integrins α1β1, α2β1, α10β1, and α11β1 (16). These receptors can be activated by several ligands such as type I collagen after recognition of its GFOGER sequence (17). α1β1 dimer was considered as the most expressed receptor in colon carcinoma (18). β1-integrin expression in tumors was correlated with reduced overall survival and reduced disease-free survival in a large cohort of CRC patients (19). Notably, β1 integrin is detected in CRC patients' serum and its level of expression appears to correlate with aggressiveness and presence of micrometastasis (20). β1 integrin overexpression is also associated with CRC progression and colorectal liver metastasis (20, 21). *In vitro*, β1 integrin expression is down-regulated in response to 3D type I collagen (22, 23). However, although β1 integrin seems to contribute to metastasis development, β1 integrin targeted therapy is not successful in CRC management. In fact, simultaneous inhibition of β1 integrin and EGFR in CRC does not improve radiotherapy efficiency (24).

Collagen also signals to cells through the receptor tyrosine kinases discoidin domain receptors DDR1 and DDR2; both of them have also been reported to interact with type I collagen (25) and to play a role in tumor progression (23). These receptors, which harbor a tyrosine kinase activity, recognize GVMGFO sequence of type I collagen (26) and exhibit a relatively late and prolonged activation (27). DDR1 is expressed in colon carcinoma and promote metastasis in invasive colon carcinoma (28–30). Concerning DDR2, a high expression was associated with higher frequencies of T4, lymph node metastasis, peritoneal spread, and worse prognosis, suggesting that DDR2 expression might be an effective therapeutic target (31).

**Figure 1:**
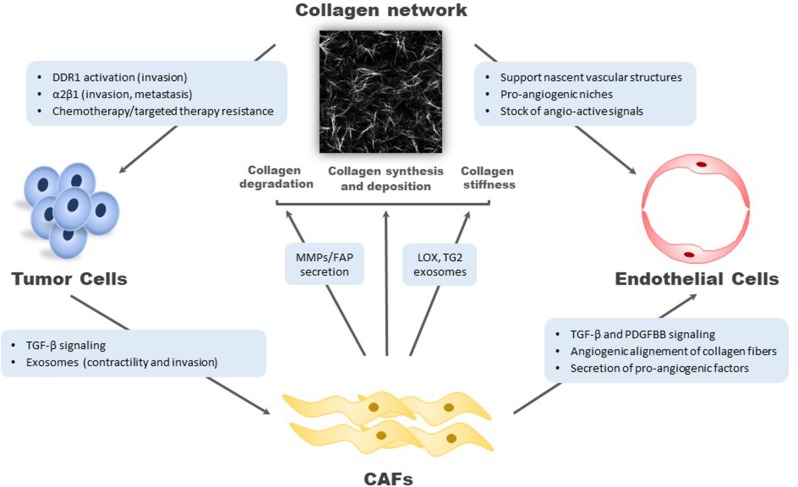
Relationship between type I collagen network and cells in tumor microenvironment. On the one hand, cancer cells, cancer-associated fibroblasts, and endothelial cells influence collagen structure and composition. On the other hand, type I collagen participates to tumor progression. DDR1, discoidin domain receptor I; FAP, fibroblast activation protein; LOX, lysyl oxidase; MMP, metalloproteinase; PDGFBB, platelet-derived growth factor BB; TG2, transglutaminase 2; TGF-β, transforming growth factor-β.

This growing data set supports a key role of collagens and their partners during tumorigenesis processes and as potential biomarkers of CRC. The following parts aim to highlight current evidence regarding the functional interplay between cells within the TME and collagen network during CRC progression. The main data are presented in Figure 1.

## The Relationship Between Collagen and Cancer Cells

Analysis of ECM signatures in patients' colon tumors has revealed that type I collagen is highly expressed (5). Accordingly, high density of type I collagen constitutes a poor prognosis factor in colon carcinoma and type I collagen-rich environment is able to induce mesenchymal gene expression and invasion (32). Beside the density, collagen topology (fiber alignment) and elasticity (stiffness) appear to be also associated to colon tumorigenesis. Brauchle et al. have demonstrated that the alignment of collagen fibers is increased in colon carcinoma tissues when compared to normal tissues and associated with increased stiffness (33). Biophysical investigations have also shown different molecular fingerprints for collagen fibers in colon carcinoma tissues when compared to normal tissues (33). Another study has shown that density and collagen fiber alignment were higher in tumor invasion front than in primary tumor and normal tissue (34). Of note, hypoxia, which is associated with collagen density and organization, has an impact on colon cancer carcinoma migration and invasion through promotion of epithelial-to-mesenchymal transition (35).

Concerning the role of type I collagen receptors in tumor progression, Roche's group has elegantly recently shown that DDR1 plays a crucial role in the invasion function of metastatic colon carcinoma (28, 29). They have particularly demonstrated that pharmacological inhibition of DDR1-BCR signaling axis decreased invasion and metastatic processes in colon carcinoma, suggesting that DDR1 targeting could be an efficient co-treatment strategy in colon carcinoma (28, 29). More recently, NSD2 circular RNA has been shown to promote DDR1 expression and CRC metastasis by targeting miR-199b-5p (36). For integrins, Wu and co-workers have lately reported that type I collagen is able to support colon carcinoma cell stemness, invasion, and metastasis through activation of α2β1 integrin heterodimer and PI3K/AKT/Snail signaling pathway (37).

Regarding the role of type I collagen in the cancer cell sensitivity to targeted therapies, a recent study has demonstrated that 3D type I collagen may protect colon carcinoma against the anti-EGFR cetuximab therapy by increasing tyrosine phosphorylation of MET and RON (32). The effect of 3D type I collagen on the sensitivity to vemurafenib of colon carcinoma, carrying the BRAF^*V*600*E*^ mutation has also been investigated. At the opposite of the general concept describing type I collagen as a shield of colon carcinoma cells against therapies, authors have demonstrated that cells seeded in 3D type I collagen were 10-fold more sensitive to the vemurafenib targeted drug. On the contrary, 3D matrix was able to protect tumor cells against the cytotoxic effect of the fluorouracil chemotherapeutic agent (38). However, another one carried out on resistance to chemotherapy and the ATP binding cassette transporter P-glycoprotein, which is encoded by *ABCB1* gene, has shown that 3D ECM is able to increase sensitivity of primary colon carcinoma cells to chemotherapy by affecting the cell polarity and consequently the polarization of P-glycoprotein expression at the cell surface (39). Interestingly, the expression of *ABCB1* gene appears to be regulated in colon carcinoma. In fact, the overexpression of the caudal-related homeobox transcription factor (Cdx2) has been reported to upregulate the expression of *ABCB1*/gene and consequently P-glycoprotein in highly resistant colon carcinoma to chemotherapy (40).

However, Cdx2 has also been described to play a role as a tumor suppressor (41). In fact, Cdx2 expression has been shown to be lower in colon carcinoma with the highest grades (42). In agreement with the role of ECM in colon carcinoma progression, type I collagen has been shown to promote tumorigenesis by downregulating Cdx2 expression (43). Brummer's group has demonstrated a few years ago a positive correlation between BRAF mutation and low level of Cdx2 expression in colon carcinoma. Type I collagen at high density has also been reported to suppress HNF4α when inducing mesenchymal gene expression *in vitro* and in patient-derived colon tumors (32). Consistently, invalidation or inhibition of HNF4α promotes colon carcinogenesis, whereas its enforced expression is able to inhibit cell growth in colon carcinoma (44, 45).

## The Relationship Between Collagen and Cancer-Associated Fibroblasts

Cancer-associated fibroblasts (CAFs) are the most abundant cell type in TME; they are an activated type of fibroblast that plays a major role in tumorigenesis and metastatic processes (46). CAFs demonstrate a functional heterogeneity in CRC that may arise from different cellular origins and can affect the clinical course of colon cancer patients (47). In CRC, an abundance of CAFs in the TME has been associated with poor outcomes and transcriptomic studies linked CAF signature with poor prognosis and highly aggressive CRC molecular subtypes. CAFs are not only associated with advanced CRC but also found in early stages (48). Several studies identified CAFs as potential prognosis and recurrence markers in patients with colon cancer (49–52). Histologic evaluations of CRC patient samples and organotypic 3D co-culture models demonstrated that CAFs are the primary drivers of collagen synthesis and remodeling in the highly desmoplastic environment found in CRC (53, 54). Interestingly, a significant heterogeneity was observed within CAF population related to collagen remodeling (55). Transcriptome and proteome profiling identified CRC CAFs as the main source for connective tissue components of the ECM, such as collagens, thus altering the molecular composition of the matrix by increasing the deposition of new matrix components (56, 57). Another way for CAFs to remodel ECM is to degrade it by using MMPs and formation of degradative protrusions. Genes induced in CRC CAFs, compared to normal colonic fibroblasts, include several tumor-promoting MMPs and TGF-β1 increased Collagen I and various proteases expression by CAFs (56–58). In CAFs, formation of invadopodia that remodel collagen fibers is dependent on twist1 and palladin (isoform 4). Twist1-expressing fibroblasts acquired CAF properties such as collagen contraction and alignment, and palladin and collagen α1(VI) were identified as two major mediators of these Twist1 effects. Interestingly, Twist1, palladin, and collagen α1(VI) are overexpressed in purified colon CAFs as compared with their normal counterparts and associate with poor prognosis in CRC (59). In addition to MMPs, CAFs also express other proteases such as the fibroblast activation protein (FAP), a collagenase and gelatinase (60). Stromal FAP expression in human colon cancer samples is a marker of early stage in cancer development and correlated with poor patient outcome (61). FAP-α activity has a strong impact on fibroblast secretome composition, including matrix processing enzymes, and influence morphology and collagen contraction capacity of immortalized CRC CAFs. Recent studies established a direct link between CAFs and the modifications of ECM organization and stiffness described in colon cancer. LOXL2, a collagen cross-linker, was reported as highly expressed in CAFs and is associated with poor CRC survival (62). Hic-5, a non-enzymatic adaptor protein, was described as a novel factor responsible for the development of CRC, by promoting in CAFs the production of collagen I and LOX that lead to stiffness of cancer tissues (63). More recently, in a collagen gel co-culture system, with fibroblasts and CRC cells, Delaine-Smith's group demonstrated that fibroblast-derived TG2 (transglutaminase-2), a protein cross-linking enzyme, induced gel stiffening by formation of thicker collagen fibers and proposed a regulatory link between TG2 and LOX. In addition, stiffness is further increased by fibroblast/CRC cross-talk and a potential role for extracellular vesicles in mediating this tumor-driven fibroblast response is suggested by authors (64). Another study reported that fibroblasts activated by late-stage CRC cell-derived exosomes became specialized in type I collagen and physical remodeling of ECM through cytoskeletal re-organization, membrane protrusion formation, and secretion of matrix-remodeling proteins (65).

## The Relationship Between Collagen and Endothelial Cells

Angiogenesis exerts crucial functions during major steps of CRC progression (1, 3, 66). Stimulation of CRC cells by oncogenic drivers such as EGF or stabilization of hypoxia-inducible factors (HIFs) was involved in the secretion of angiogenic diffusible factors and ECM structural compounds in the TME (67). Moreover, collagen supports nascent vascular structures during intussusceptive angiogenesis in CRC (68). A nine-gene signature including collagen I, X, and XI was specifically enriched in angiogenic and hypoxic CRC gene sets (4). Another study identified a matrisomal signature of 110 genes induced during the angiogenic switch of the standard RIP1-Tag2 murine model of tumor angiogenesis (3). The expression of this set of genes, which includes collagens I, VI, VIII, and X and various ECM regulators was positively correlated with that of endothelial cell markers and increased with CRC progression. This signature was also specifically induced in hepatic metastasis suggesting a functional contribution to both early events and metastatic cascade. It is now well-established that tumor and stromal cells synergize to activate pro-angiogenic signals in the TME (3, 61, 66, 69, 70). CAFs and tumor-associated macrophages (TAM) are both involved in TGF-β signaling activation during the angiogenic switch (3). Stromal activation of this pathway promotes both tumor initiation and early metastatic events (66) and was specifically associated to consensus molecular subtypes CMS4 of CRC that express various angiogenesis markers and present the worst overall survival (71). Several reports illustrated the contributions of tumor-resident or infiltrated stromal cells to ECM-modifying events that accommodate endothelial cells' fitness and provide angiogenic cues (61, 69, 72). Although a clear scenario is sometimes difficult to draw on the angiogenic consequences of collagen deposition, emerging angio-active parameters include types of collagens (network, fibrils-anchoring or fibrillar collagens that convey different angiogenic signals), topology, and stiffness. Post-translational modifications such as proteolytic degradation or cross-linking can modulate the biophysical properties of collagen-rich scaffolds (11, 70, 72, 73). FAP-α expression and activity were linked to the secretion of pro-angiogenic factors such as angiopoietin-1 and VEGF-C by colon patient-derived CAFs (61). Gain- and loss-of-function experiments illustrated that FAP-α-dependent CAF secretome can stimulate 3D endothelial spheroids sprouting. *In vivo*, targeting of FAP-α into an immune-competent murine model of colon cancer decreased blood vessel density and induced fibrillar collagen accumulation (69). The activity of SNAI1 and PDGFBB contributes to CAF ability to assemble aligned collagen fibers that promote endothelial cell proliferation and morphogenesis in a 3D model of CAF-derived matrices (72). SNAI1 expression by fibroblasts was also associated with the abundance of CD34-positive endothelial cells in an *in vivo* model of CRC. A pro-tumoral action of TAM could be explained by their ability to assemble collagenous ECM enriched with type I, VI, and XIV collagens (70). These fibers were deposited, cross-linked, and linearized at areas of tumor invasiveness demonstrating the crucial importance of TAM in organizing collagenous ECM niches. Co-culture of TAM with CRC cells can potentiate the production of tumor-derived MMP2 and MMP9 (74). Recruitment of collagenolytic enzyme-expressing immune cells in the CRC TME might influence the bioavailability of ECM-immobilized angiogenic factors such as VEGF, as reported in other tumor context (75, 76). Collagen-enriched niches emerge as biomarkers of desmoplastic and angiogenic CRC microenvironment (77, 78). High expression of collagens I and IV, with tumor endothelial marker-1 (TEM-1, endosialin), especially when distributed around tumor vessels, allows stratification of CRC patients according to their poor prognosis (77). Collagen-enriched niches might also account for the adaptive response of the TME to anti-angiogenic therapies (78, 79). Collagen IV empty sleeves resulting from tumor vessel pruning triggered by VEGFR2 can promote a rapid vascular regrowth after treatment withdrawal (79). VEGFR2 blocking in a CRC model normalized tumor vessels, decreasing diameter while ameliorating collagen IV perivascular coverage (78). Endostatin, a collagen XVIII-derived fragment, is an inflammatory marker detected around blood vessels and in the plasma of advanced CRC patients (12). This molecule, efficient to inhibit both lymphangiogenesis and hemangiogenesis (80), is considered as a valuable tool to control metastatic CRC growth since several studies reported its moderate toxicity without observing the increased metastatic dissemination encountered in response to the anti-VEGF antibody bevacizumab (30).

## *In vitro* TME Models Using Collagen

Two-dimensional (2D) collagen-coated systems' routine use has largely shown their limitations to summarize the complexity of tumor initiation and progression processes. It is absolutely necessary to include some major extracellular components to mimic properties of the TME such as the spatial configuration (81) and the addition of supporting materials with mechanical properties close to the ECM encountered during disease progression (82). The use of *in vitro* 3D models should fill the gap between traditional 2D cell culture and animal models, by mimicking the cancer micro- and macro-environment potentially able to integrate multiple cell types in a controlled environment, and should allow one to better characterize CRC drivers and develop new therapeutic strategies in constantly upgraded models of growing complexity. One possible approach is to develop spheroids of cancer cells seeded on low-attachment tissue culture plates. Whereas, this type of culture allows cancer cells to communicate with one another and to release low levels of intrinsic collagen (83), substantial aspects of TME are still missing. More complex models using biological scaffolds such as collagen are therefore added to create an ECM to obtain biomimicry and study cancer progression by recreating the TME. Patient-derived xenograft models are an important tool for preclinical and clinical research, especially when orthotopically transplanted. However, in this model, the principal limit is that TME cannot be properly reconstituted owing to important stromal cells such as cancer-associated fibroblasts and endothelial cells, which are not derived from the tumor samples and can be late recruited (84). New 3D models of cancer using a collagen matrix can promote the crosstalk between cancer and stromal cell. Co-cultures of different CRC cell lines with fibroblasts and endothelial cells in 3D spheroids have been elaborated to test drug dose response and compared with results in 2D and homotypic 3D cultures. The results suggest that 3D co-cultures are more relevant, providing a higher level of translational information that should help to define patient-specific treatment options (85).

Pape and colleagues developed a CRC model using high-density type I monomeric collagen, termed tumoroids (86). This model consists of a central cancer mass containing either the highly invasive HCT116 or less invasive HT29 cells embedded in collagen type I hydrogels to mimic the TME *in situ* (87). The stromal compartment in this model is easily manipulated and ECM components and stromal cell types can be added accordingly. Furthermore, on-chip biomimetic microenvironments using microfluidic technologies are being developed to better reproduce the complexity of *in vivo* restrictions. In this model, human colonic microvascular endothelial cells cultivated in a 3D vessel-mimetic device are attached to the wall of the lateral channels of the microfluidic chip whereas HCT-116 cells are embedded in collagen IV-enriched Matrigel in the central chamber (88).

Considering the feature of tumor heterogeneity, the main limitation of these models is the presence of a single CRC cell type exhibiting a unique genetic pattern. The development of more realistic preclinical models is absolutely required and is a major challenge for the coming years, especially for improving drug screening. The use of patient-derived 3D tumor models may provide a solution to overcome the oversimplified 2D cell cultures and the limitations of *in vivo* models (89). These new designs are not intended to completely supplant but rather complete and expand the currently available techniques.

## Author Contributions

All authors listed have made a substantial, direct and intellectual contribution to the work, and approved it for publication.

## Conflict of Interest

The authors declare that the research was conducted in the absence of any commercial or financial relationships that could be construed as a potential conflict of interest.

## Abbreviations

CAF: cancer-associated fibroblast
CRC: colorectal cancer
DDR: discoidin domain receptor
ECM: extracellular matrix
FAP: fibroblast activation protein
SHG: second harmonic generation
TME: tumor microenvironment.
